# Monoclonal antibody against EV71 3D^pol^ inhibits the polymerase activity of RdRp and virus replication

**DOI:** 10.1186/s12865-019-0288-x

**Published:** 2019-01-22

**Authors:** Yaoming Li, Jie Yu, Xiuwen Qi, Huimin Yan

**Affiliations:** 1 Mucosal Immunity Research Group, State Key Laboratory of Virology, Wuhan Institute of Virology, Chinese Academy of Sciences, Wuhan, 430071 People’s Republic of China; 2 University of Chinese Academy of Sciences, Beijing, 100049 China

**Keywords:** Monoclonal antibody, Enterovirus a 71, RdRp, Antiviral

## Abstract

**Background:**

Enterovirus A 71 (EV71) is a neurotropic virus that may lead to acute flaccid paralysis, encephalitis, cardiopulmonary failure or even death. No vaccine and defensive drug controlling EV71 is currently available, novel and efficient antiviral drug or vaccine is therefore urgently needed. 3D^pol^ (RNA-dependent RNA polymerase (RdRp)) has been an important target for anti-EV71 drug development.

**Methods:**

A panel of monoclonal IgG antibodies (mAbs) against EV71 3D^pol^ were generated by traditional cell fusion methods. And the antibody affinity and specificity to EV71 3D^pol^ were evaluated by Enzyme-linked Immunosorbent Assay (ELISA), Indirect Fluorescent Assay (IFA) and Western blotting. Antiviral activities of these antibodies were also determined in vitro and in vivo.

**Results:**

Two mAbs towards EV71 3D^pol^ were able to effectively suppress EV71 replication in Vero-1008 cell when intracellarly delivered. And they also dampened the RNA polymerase activity of 3D^pol^ in vitro. More importantly, these mAbs provided partial protection in EV71-challenged neonatal murine challenge model.

**Conclusions:**

These results showed that two of mAbs against EV71 3D^pol^ inhibited EV71 replication and could be utilized as promising therapeutic drug candidate.

**Electronic supplementary material:**

The online version of this article (10.1186/s12865-019-0288-x) contains supplementary material, which is available to authorized users.

## Background

Enterovirus A 71 (EV71) mainly infects children under the age of 5 years old, occasionally causing damage in neuron system [1]. Sporadic cases of EV71 infection appear throughout the world, primarily in Southeast Asian countries [2]. As the major causative agent of hand, foot and mouth disease (HFMD), EV71 contains a single plus-strand RNA genome of approximately 7 kb in length. During viral infection, four capsid proteins (VP1, VP2, VP3 and VP4) are translated, among which VP1 plays dominant role during viral acquisition and dissemination. While viral non-structural proteins (2A^pro^, 2 BC, 2B, 2C, 3AB, 3A, 3B (VPg), 3CD^pro^, 3C^pro^, and 3D^pol^) manage immune escape, precursor protein cleavage, and progeny virus maturation [3]. Particularly, 3D^pol^ directs uridylylation of 3B and replication of viral genome RNA [4].

Currently, no specific vaccine or drug is available for preventing EV71 infection. Monoclonal antibody (mAb) has been demonstrated promising for preventing from virus infection [5]. Many EV71-neutralizing murine mAbs have been generated by hybridoma technique using SP70 peptide [6], inactivated virus particle [7], live virus [8], or recombinant virus-like particles (VLPs) [9] as immunogens. However, all the above mentioned mAbs are specifically against viral capsid protein VP1. Few mAbs against non-structural protein of EV71 has been studied in detail. Our recent study on throat swab specimens from clinically confirmed EV71-infected out-patient children showed the antibody response after EV71 infection was not only against viral structural proteins such as VP1 and VP2, but also against non-structural protein 3D^pol^. Furthermore, the EV71 infection of the children correlated with their preexisted IgG against EV71 induced in former infection [10]. We wondered whether the antibody against non-structural protein 3D^pol^ in the course of EV71 infection has antiviral function. Here in this report, we developed a panel of EV71 3D^pol^-specific mAbs, and characterized their antiviral activities in vitro and in vivo.

## Results

### Generation of mAbs against 3D^pol^ of EV71

**Fig. 1:**
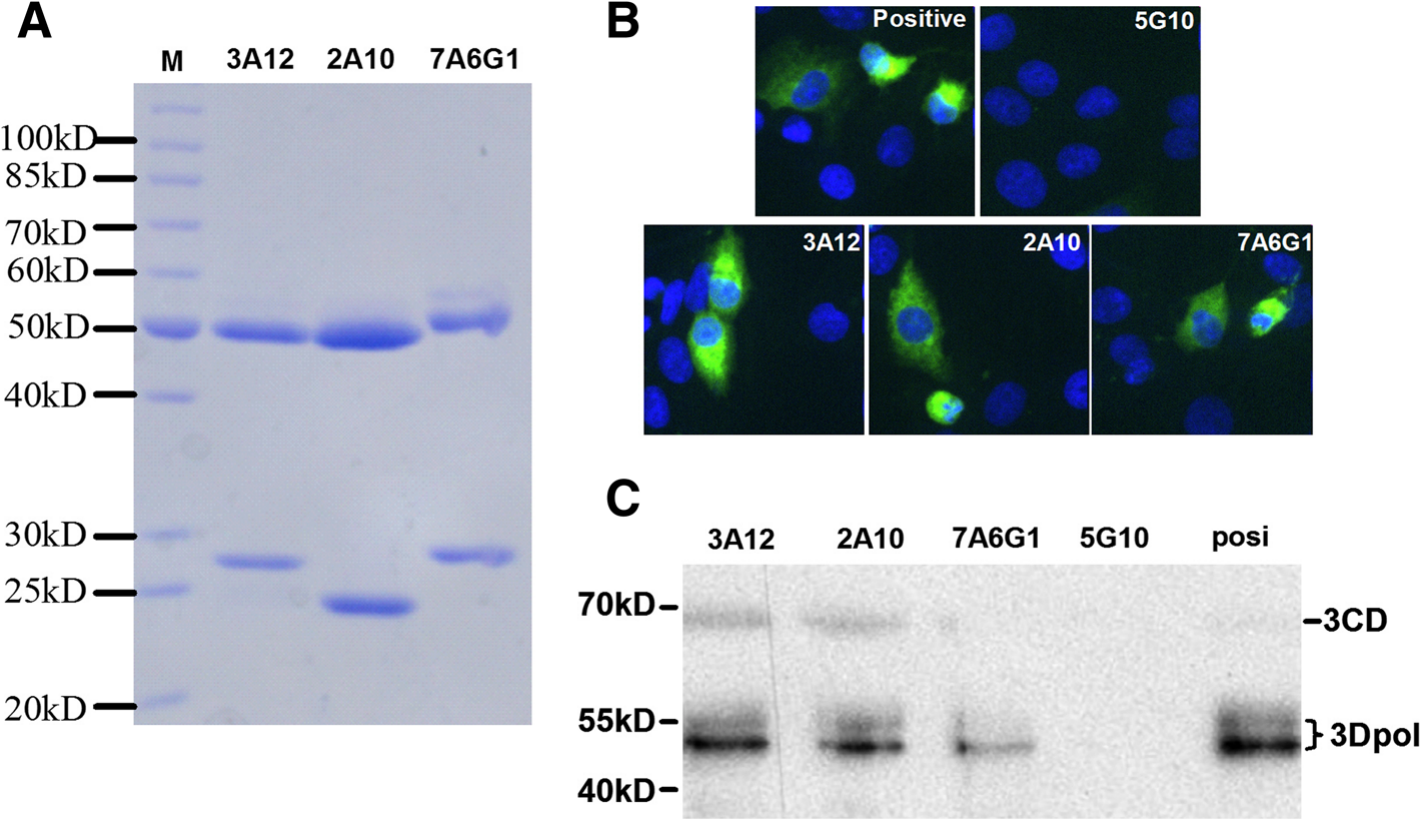
Characteristics of IgG mAbs against EV71 3D^pol^. **a** SDS-PAGE for mAbs 3A12, 2A10, 7A6G1. **b** Reactivity of indicated mAbs (3A12, 2A10, and 7A6G1) with EV71-expressed 3D^pol^ by IFA. **c** Western blot for mAbs IgG (lane 1, 3A12; lane 2, 2A10; lane 3, 7A6G1; lane 4, 5G10; and lane 5, positive antibody control). 3D^pol^-immunized mice serum was used as positive antibody control. MAb 5G10, which was specific for Salmonella flagellin, served as negative antibody control

We generated mAbs against 3D^pol^ by traditional hybridoma method and obtained a panel of mAbs against EV71 3D^pol^. We achieved three mAbs 3A12, 2A10 and 7A6G1, and the property of each mAb was summarized in Table 1. 3A12 and 7A6G1 are IgG1 subtype with typical heavy chains about 50 kD and light chains about 27 kD, while 2A10 is IgG2a subtype with typical heavy chains about 50 kD and light chains about 24 kD (Fig. 1a). Ratio of each mAb heavy chain(HC)/light chain(LC) was close to 2 (Additional file 1: Figure S1). Specificities of the mAbs were identified with EV71-infected Vero-1008 cells by IFA using the mAbs as primary antibodies. The serum from 3D^pol^-immunized mice was used as positive control here. The mAb 5G10 which is specific to *Salmonella* flagellin severed as negative mAb control [11]. The result showed the EV71-specific green florescent signals were mostly located in cytoplasm (Fig. 1b). Mean fluorescence intensity (MFI) of each virus infected cell was about 5000 (Additional file 2: Figure S2). Cell lysate of EV71-infected Vero-1008 cell was subject to Western blotting assay, a clear 51 kD band of 3D^pol^ and a 55 kD band of cleaved 3CD^pol^ protein could be detected, while a 70 kD band of 3CD^pol^ (a precursor of 3D^pol^) could also be detected (Fig. 1c) as similar as reported previously [12].

**Table 1:** Charateristics of mAbs IgG against EV71 3D^pol^

mAbs	IgG subtype	Recognized region (AA)	ELISA	IFA	Western blot
3A12	IgG1	1–120	+++	+++	++
2A10	IgG2a	121–250	+++	+++	++
7A6G1	IgG1	360–462	+++	+++	+

*AA* amino acid

### Identification of the regions recognized by the mAbs

**Fig. 2:**
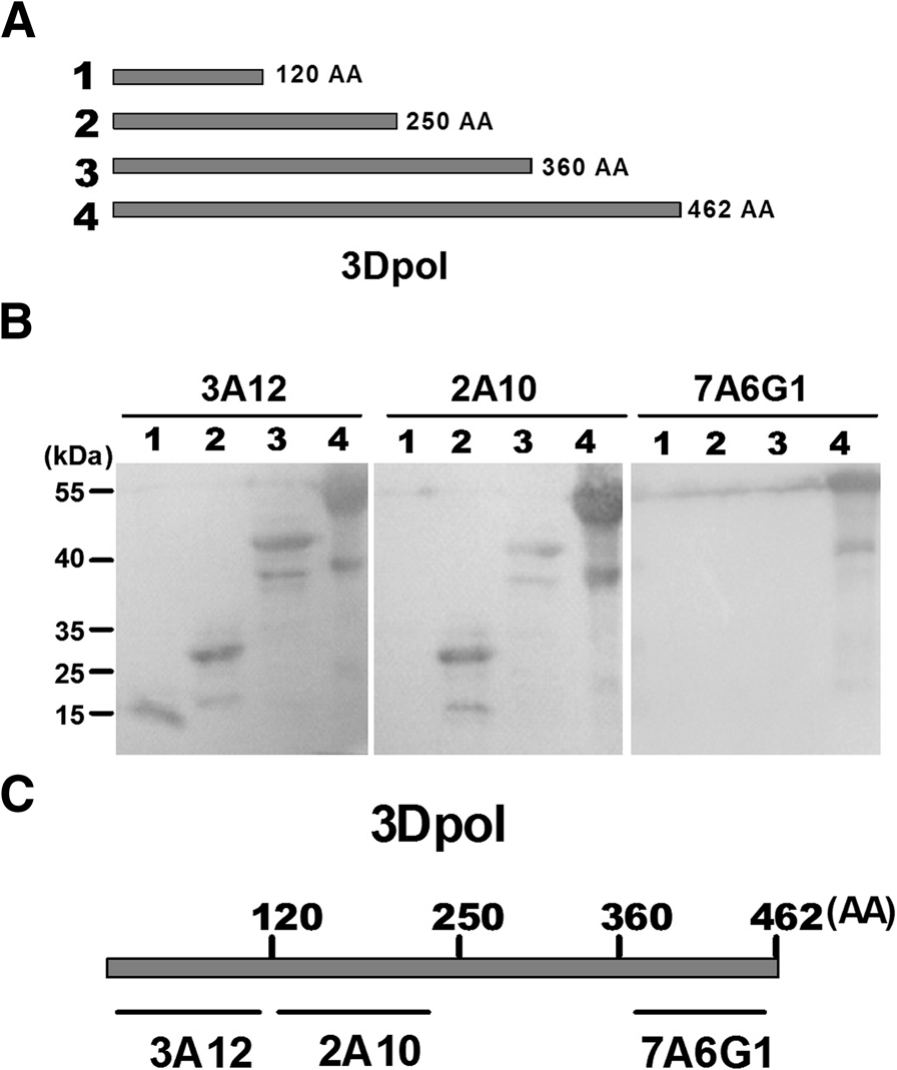
Identification of regions recognized by 3D^pol^-specific mAbs. **a** The intact 3D^pol^ protein and three C-terminal truncated proteins of 3D^pol^ were shown in the schematic diagram. **b** Truncated 3D^pol^ were used to identify mAb domains by using Western blot. **c** 3A12-recognized region covers 1–120 amino acid of 3D^pol^, 2A10 in 120–250, and 7A6G1 in 360–462, respectively

To identify the recognized epitopes recognized by the distinct mAbs, three recombinant 3D^pol^ proteins with serial C-terminal truncations were prepared for Western blotting assay (Fig. 2a). 3A12 showed binding ability to all the three truncated proteins and the intact 3D^pol^ protein. 2A10 showed binding ability to all the proteins but truncated in C-terminal 120–462 amino acid, while 7A6G1 to none of the three truncated proteins but only the intact 3D^pol^ (Fig. 2b). The result suggested that the 3A12 recognized 3D^pol^ in 1–120 amino acid of 3D^pol^ N-terminal, 2A10 recognized in 120–250 amino acid, and 7A6G1 recognized in C-terminal 360–462 amino acid (Fig. 2c).

### In vitro inhibition abilities of the mAbs

**Fig. 3:**
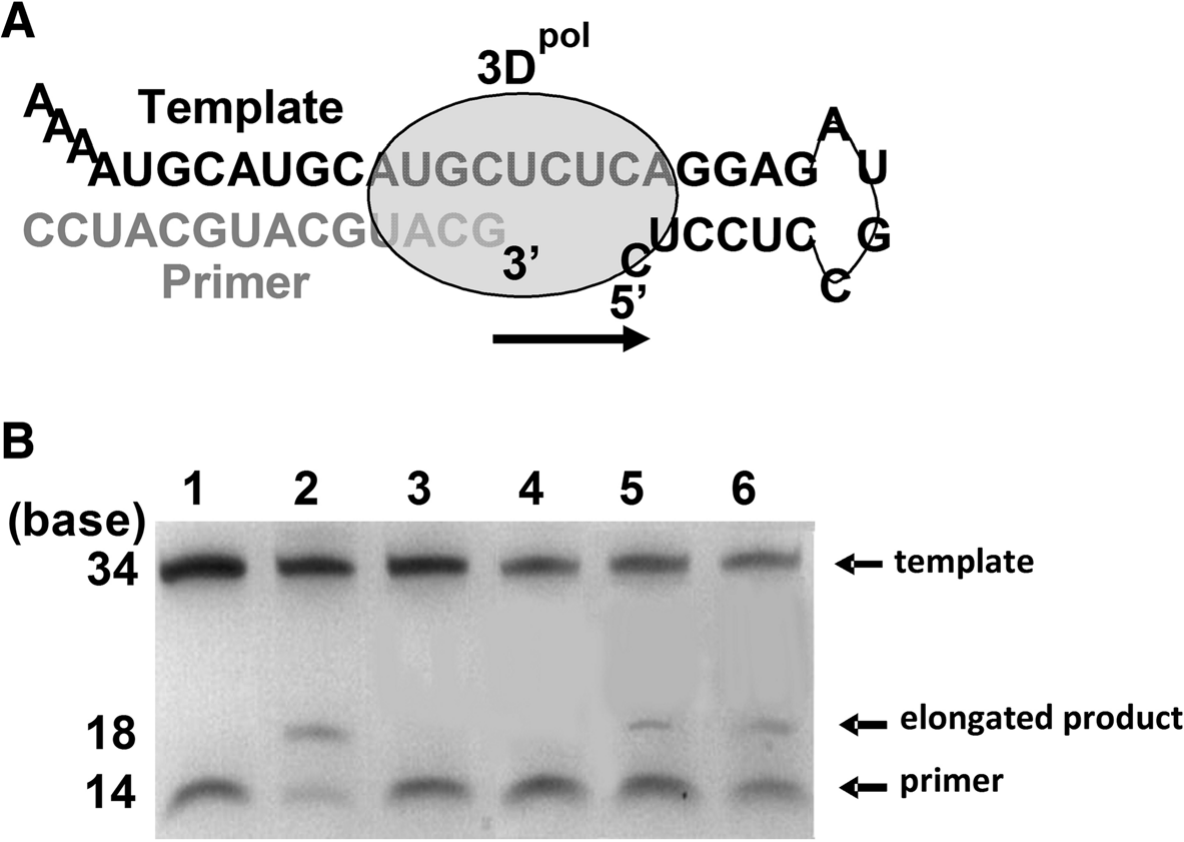
mAbs suppressed 3D^pol^ activities of RNA elongation. **a** Schematic diagram of RdRp-mediated RNA elongation. **b** Application of mAbs against 3D^pol^ suppressed the RNA elongation. Lane 1, no polymerase control; lane 2, positive control; lane 3, 3A12; lane 4, 2A10; lane 5, 7A6G1; lane 6, 5G10. Three independent experiments were performed, and the representative data were presented

Inhibition abilities of the mAbs were rapidly evaluated in an RdRp-mediated RNA elongation system (Fig. 3a), in which antibody binds to RdRp, the RNA elongation would be blocked. When mAbs 3A12 or 2A10 engaged with 3D^pol^, the elongated RNA band almost disappeared, however the applications of 7A6G1 and 5G10 did not significantly inhibit the quantity of elongated RNA (Fig. 3b). 5G10 served as an irrelative antibody control. Therefore, mAbs 3A12 and 2A10 remarkably inhibited the RNA elongation compared with 7A6G1 and 5G10.

### Antiviral capacity of the intracellular mAbs

**Fig. 4:**
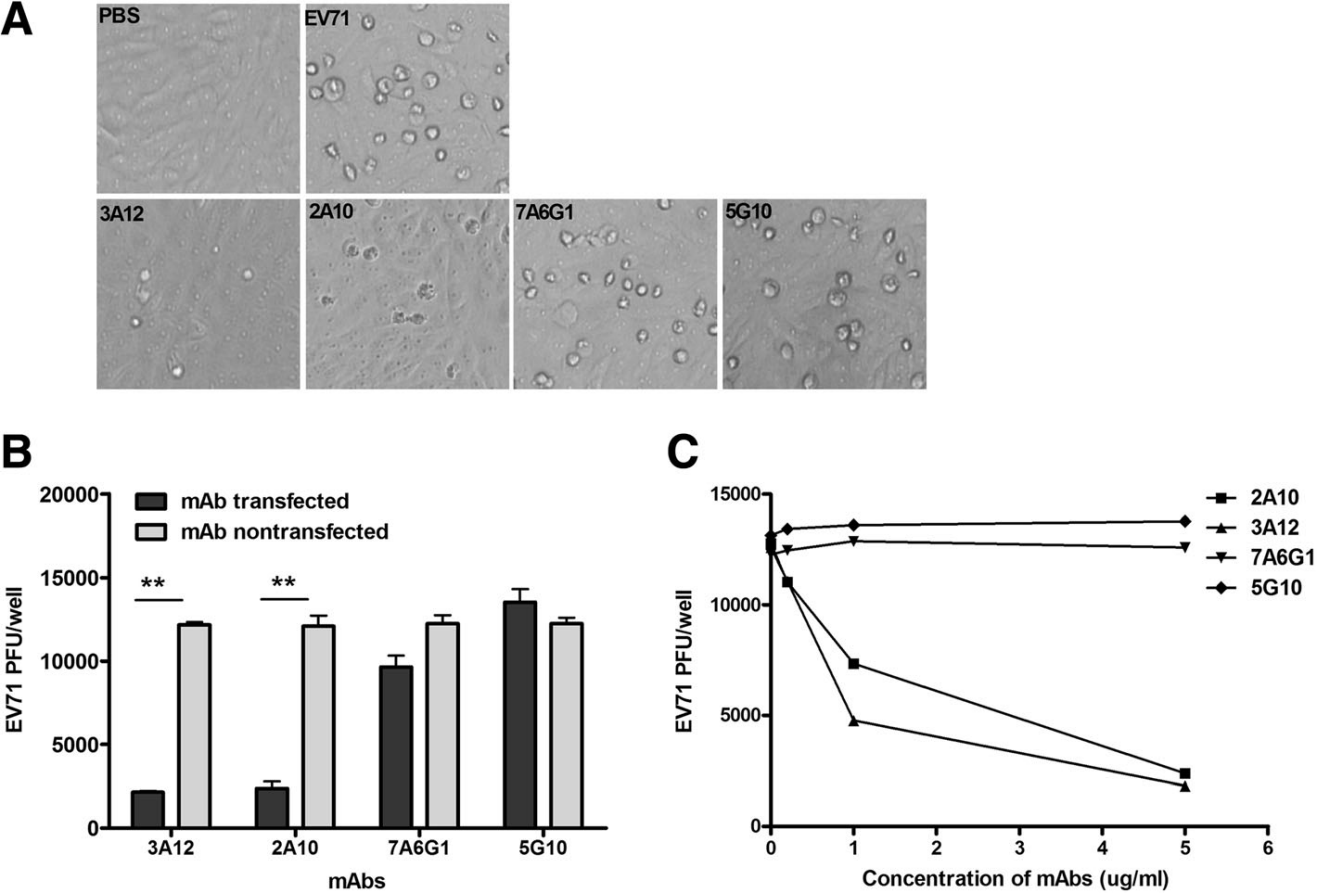
MAbs intracellularly inhibit EV71 replication. **a** Representative CPE on Vero-1008. **b** Indicated mAbs 3A12 and 2A10 inhibit EV71 replication when intracellularly delivered. **c** Indicated mAbs intracellularly inhibiting EV71 replication is dose-dependent. Three independent experiments were performed in duplicate, and the representative data were presented

In order to demonstrate whether intracellular mAbs could inhibit viral replication, antiviral capacity of mAbs were evaluated in EV71-infected cell. It was observed that over 80% cell were transfected with mouse IgG upon DOTAP (data not shown). After transfection with mAbs (3A12, 2A10, 7A6G1 or 5G10), EV71-infected cells were taken photographs at 24 h, it was observed that 3A12 and 2A10 resulted in less CPE when compared with 7A6G1 and 5G10 (Fig. 4a). And the EV71-infected samples were subjected to test the viral titer. Among those applied antibodies, 3A12 or 2A10-transfected Vero-1008 produced about 2000 PFU of EV71 per well, which was significantly lower than 7A6G1 or 5G10-transfected cells (*p* < 0.01) (Fig. 4b). However, without the application of DOTAP both the 3A12 and 2A10 could not suppress the production of EV71 (Fig. 4b).

Furthermore, it was demonstrated whether antibody-mediated intracellular inhibition is dose-dependent. Based on the same method, EV71-infected Vero-1008 were transfected with mAbs 2A10 or 5G10 at increasing dose, subsequently the viral production decreased accompanying the increasing quantity of mAb 2A10 (Fig. 4c). Without transfection process, 3A12 and 2A10 could almost not interrupt viral propagation (Additional file 3: Figure S3). In summary, 3A12 and 2A10 intracellularly neutralize 3D^pol^ and inhibit viral replication in a dose-dependent manner.

Our IFA results also showed that majority of 3D^pol^ proteins (RdRp) located in cytoplasm and nuclear of EV71-infected cells (Fig. 1b). Extracellular 3A12 and 2A10 with molecular weight about 150 kD were barely able to pass through the cell membrane into cytoplasm in vitro. Only by intracellularly delivering by using protein transfection reagent DOTAP could 3D^pol^-specific mAbs exert antiviral function.

### An in vivo evaluation of antiviral efficacy of the mAbs

**Fig. 5:**
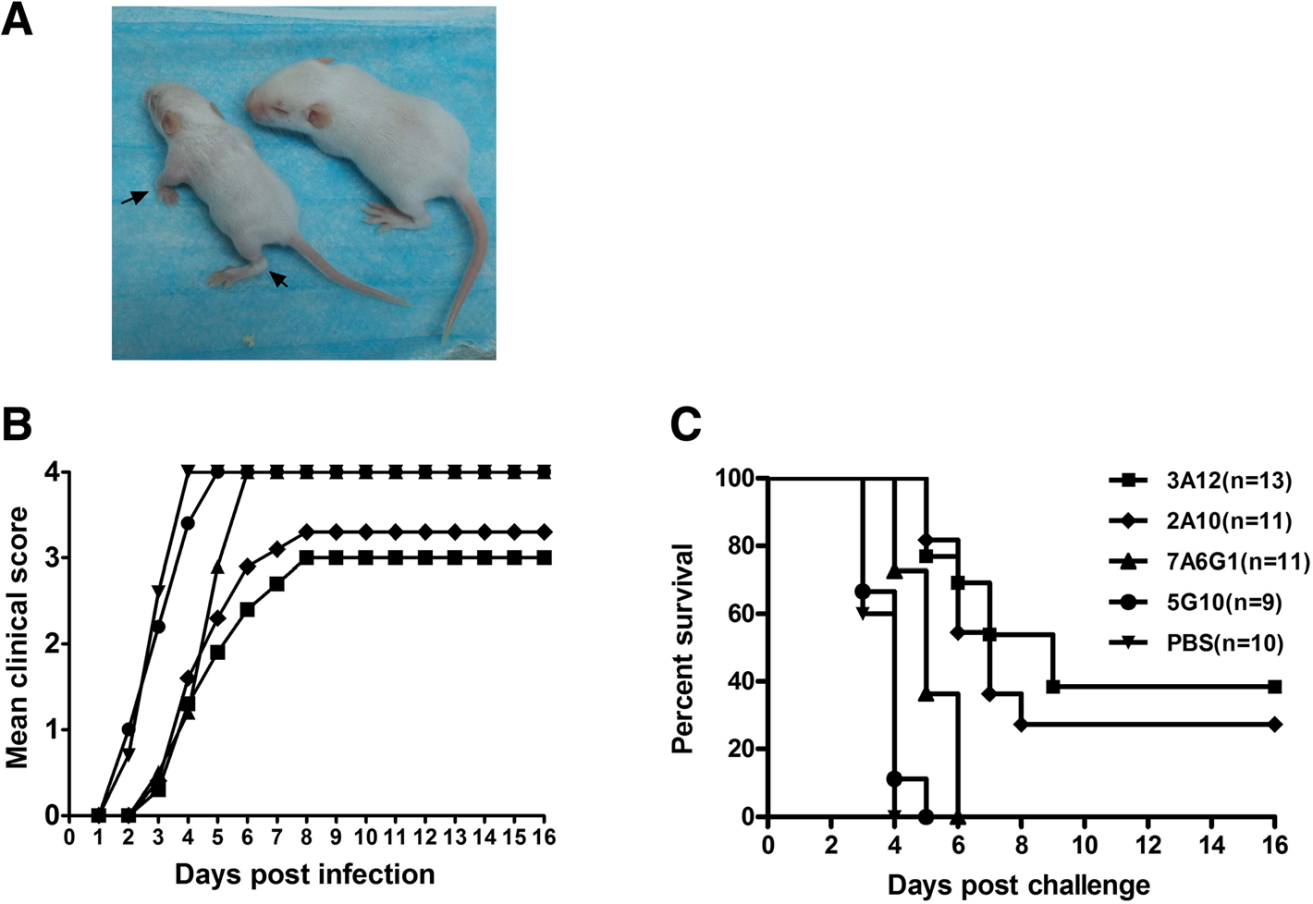
Kaplan-Meier analysis of antiviral efficacy of EV71 3D^pol^-specific mAbs on ICR neonatal mice. **a** A neonatal mouse infected with EV71/MAV-VR exhibited limb paralysis (indicated by arrows). The virus inoculated mice were monitored daily for (**b**) clinical score and (**c**) survival for a period of 16 days. Clinical scores were graded as follows: 0, healthy; 1, reduced mobility; 2, limb weakness; 3, paralysis; 4, death. The number of mice in each group were indicated in one bracket

To identify whether the 3D^pol^-specific mAbs could exert antiviral function in vivo*,* neonatal ICR mice i.p. received mAbs and were then challenged as described in Methods. Briefly, five ICR mice with 12 days’ pregnancy were purchased and housed individually. At that time, each pregnant mouse was randomly allocated to different treatment groups (3A12, 2A10, 7A6G3, 5G10, and PBS) in a single-blind manner. Subsequently, 13, 11, 11, 9, and 10 neonatal mice were given birth in the corresponding broods respectively. Therefore 54 neonatal mice were totally delivered and included in the in vivo study. After challenge, all the neonatal mice in control group develop typical EV71 clinical syndrome including limb weakness and paralysis (Fig. 5a and b) which were calculated in a double-blind manner as previously described [11]. After observation for 16 days, five and three mice still survived in those groups received injection of 3A12 and 2A10, respectively. Thus 3A12 (5/13, 38.5%) and 2A10 (3/11, 27.3%) provided significantly higher protection efficacy then 5G10 (0/9, 0%) (*p* < 0.01) (Fig. 5c). Interestingly, although 7A6G1 finally did not confer protection for mice, it prolonged the survival time for the challenged mice.

## Discussion

Our earlier clinical data showed that EV71-infected cohorts had IgG antibodies against both structural and non-structural protein. It was not known that whether the IgG against viral polymerase 3D^pol^ might exert antiviral activity. In the current study, we focused on neutralization capacity of the mAbs against the 3D^pol^ and its possible mechanism for neutralization in vitro.

VP1,VP2, and VP3 jointly form a heterologous trimer, which plays critical role in the early step of virus attachment during the viral life circle. VP4 is the smallest capsid protein embedded in viron, which therefore are believed not likely to be involved in virus attachment process. Among the three outer capsid proteins, VP1-induced antibody was most likely associated with immune protection against EV71 infection. In addition, it was revealed that VP1-derived SP70 peptide was able to elicit antibody-associated immune protection. However, there appeared very few reports regarding VP2, VP3 and VP4-induced immune protection for EV71infection.

The antibodies exert antiviral function presumably through distinct mechanisms. Firstly, antibody suppresses EV71 replication probably via disturbing polymerase activity of 3D^pol^. EV71 3D^pol^ (RdRp) directs novel viral RNA genome elongation and duplication. There are several functional domains on 3D^pol^, including template RNA entrance tunnel, catalytic domains for adding NTP, catalytic domains for uridylylation of 3B, NTP entrance tunnel, and even the sumolyzation domains [13]. Masking any of the above domains may block the function of 3D^pol^, suppress RNA genome replication and even inhibit viral reproduction. The mAbs 3A12 and 2A10 binding to 1–250 amino acids (Fig. 2c) of 3D^pol^ possibly interrupted the polymerase on important domains. Secondly, the Fc portion of IgG could participate in the antibody-mediated neutralization in vivo. Li et al., demonstrated the neonatal FcRn receptor is critical for protection efficacy of IgG against HSV-1 infection in mouse model [14]. FcRn-KO mice significantly decrease the protection efficacy when challenged. While FcRn is an IgG Fc domain-specific receptor in most epithelial cell managing IgG transcytosis. Thus the IgG Fc fragement is considered essential in the in vivo protection of the mAbs. In another example, intracellular TRIM21 (an E3 ligase) was demonstrated as a crucial molecule for neutralizing virus. TRIM21 is an intracellular Ig Fc receptor including IgM, IgG and IgA [15]. Therefore, IgG Fc should be involved in the antiviral process through distinct mechanisms in vivo. The mechanisms of in vivo antiviral activity of antibody likely included, but not lest, antibody-dependent cell-mediated cytotoxicity (ADCC) and intracellular 3D^pol^ activity inhibition. Ebola virus GP-specific mAb was not able to inhibit virus infection in vitro, however showed significant antiviral capacity in vivo, indicating multiple underlying mechanisms. Our data showed 7A6G1 was able to bind EV71 3D^pol^ (Fig. 1), but failed to prohibit 3D^pol^ polymerase activity in RNA elongation in vitro (Fig. 3). Therefore we deduced in vivo 7A6G1 ADCC effect provided transiently suppression of EV71 replication, rather than complete viral clearance. Actually, the exact in vivo mechanism by which 3D^pol^-specific mAbs neutralize the RdRp and suppress the viral replication needs further verification and elucidation.

The mAbs could become novel drug prototype for treating EV71 infection. Several literatures reported the potential applications of anti-3D^pol^ small molecule drugs. For instance, Ribavirin (1-β-D-ribofuranosyl-1,2,4-triazole-3-carbox-yamine) is a conventional nucleoside analogue that targets the RdRp of Picornaviruses with relatively higher IC_50_ 266 μM [16]. Recently, a piperazine-containing pyrazolo pyrimidine derivative, DTriP-22, and aurintricarboxylic acid (ATA), were identified to inhibit the viral replication by specifically interfering 3D^pol^ function of RNA initiation and elongation [17, 18]. Since each of the mentioned small molecule drugs for EV71 caused cell damage at dosage used, it could be a concerned issue for further application of those small molecule drugs. Our result showed 3D^pol^-specific antibody could strikingly suppress the virus replication in vitro, but without cell damage at applied concentration (data not shown), therefore it implied the antibodies had safety advantage in anti-EV71 drug development. Actually, 3D^pol^ has been considered as a critical target for anti-EV71 drug development in recent years. Single-chain antibodies against a plant viral RNA-dependent RNA polymerase confers virus resistance [19, 20]. Cell penetrable human monoclonal ScFv specific to NS5B polymerase (RdRp) interferes of HCV replication [19, 20]. Therefore, upon the modification and/or humanization these mAbs could be more potential candidate fighting virus infection.

Taken together, the mAbs (3A12 and 2A10) bind to 3D^pol^ protein and inhibit its RNA elongation activity in vitro, and most importantly we demonstrated their antiviral efficacies in EV71-challenge neonatal mice model. It implies that modified antibodies (3A12 and 2A10), such as scFv, murine-human chemeric or humanized IgG, could be used as a promising therapeutic drug, more importantly the 3D^pol^ may also be a potential anti-EV71 vaccine component.

## Conclusions

EV71 3D^pol^-specific antibodies showed antiviral activities in vitro and in vivo. Thus antibody against nonstructural protein could be utilized as potential therapeutic drug after rational design and optimization.

## Methods

### Cell, virus, antibody and protein

Vero-1008 was grown and maintained in Dulbecco’s Modified Eagle’s Medium (DMEM, Sigma-Aldrich, MO, USA) supplemented with 10% heated-inactivated fetal bovine serum (Gibco, Life Technologies Corp., Calif., USA), 100 μg/ml streptomycin and 100 IU/ml penicillin (Sigma-Aldrich, MO, USA) at 37 °C with 5% CO_2_. A BrCr strain of EV71 was provided by Dr. H Wang in Wuhan Institute of Virology (WIV), Chinese Academy of Sciences (CAS). A mouse-adapted EV71 (EV71/MAV-VR) used in this study was kindly provided by Dr. Z Huang in Key Laboratory of Molecular Virology and Immunology, Institut Pasteur of Shanghai, Shanghai Institutes for Biological Sciences, CAS, China [21]. The BrCr and MAV-VR EV71 strains have the same 3D^pol^ amino acid sequence. For clarification, EV71/MAV-VR was only used in the challenge experiment, while the rest EV71 mentioned in this study represent EV71 BrCr strain. A purified recombinant 3D^pol^ protein was provided by Dr. P Gong in WIV, CAS [22].

### Animals

BALB/c aged 6–8 wks and pregnant ICR mice were purchased from Beijing Laboratory Animal Research Center and housed under specific pathogen free (SPF) conditions in the Animal Center of WIV, CAS, China.

### Generation of mAbs against EV71 3D^pol^

IgG mAbs against EV71 3D^pol^ were developed by traditional hybridoma technique as previously described [23]. In brief, 5-wk-old female SPF BALB/c mice were immunized subcutaneously with 100 μg of 3D^pol^ at 2-wk interval. Four wks after the last booster and 3 days before cell fusion, the mice were boosted with 200 μg of 3D^pol^. Three days later, murine splenocytes were harvested and fused with SP2/0 using 50% polyethyleneglycol (Sigma-Aldrich, MO, USA). Hybridoma culture supernatants were preliminarily screened by EV71 3D^pol^ protein using ELISA. The positive hybridoma cells were cloned by a limiting dilution and the stable hybridoma clones were injected into liquid paraffin-pretreated abdominal cavities of BALB/c mice. Subsequently, the mAbs were harvested and purified from the ascite with an antibody purification kit according to the manufacturer’s specifications (NAb™ Protein A/G Spin Kit, Thermo Scientific, IL, USA). This mouse study was approved by the ethics committee of life science and research in Wuhan Institute of Virology (WIV), Chinese Academy of Sciences (CAS) (No. WIVA09201502).

### Immunofluorescence assay (IFA)

Vero-1008 cells were seeded into 24-well tissue culture plates (Costar Corning Inc., NY, USA) at a concentration of 1 × 10^5^ cells per well. When the cells reached approximately 80% confluence, culture medium was removed and then cells were washed three times with sterile PBS (pH 7.4) and incubated with EV71 (MOI = 0.1) for 1 h at 37 °C. After removal of supernatant, fresh medium was added and cultures were incubated at 37 °C. At 24 h of post infection, the infected cells were fixed with absolute methanol and processed for indirect immunofluorescence assay (IFA) using purified mAbs, followed by fluorescein isocyanate-conjugated goat anti-mouse IgG for confirming antibody specificity against EV71. Fluorescent images were examined under a fluorescent microscope.

### Immunoblotting

The EV71-infected Vero-1008 were separated by SDS-PAGE and then electrophoretically transferred to PVDF membrane (GE Healthcare, PA, USA). The membrane was blocked for 1 h at RT with blocking solutions containing 5% Bovine Serum Albulmin (BSA) in TBS (20 mM Tris-HCl (pH 7.5), 150 mM NaCl), and then incubated overnight with purified mAbs at 4 °C. After washing with T-TBS (TBS + 0.05% Tween 20), the membrane was incubated for 45 min with HRP-conjugated anti-mouse IgG (Jackson ImmunoResearch Laboratories, PA, USA). After washing with T-TBS, the membrane was developed by treatment with ECL Western Blotting Detection Reagents (Boster, Wuhan, China). A house keeping protein β-actin was also detected as control.

### EV71 infection and mAbs IgG transfection

Vero-1008 cell was seed in 24-well plate 1 × 10^5^ cell per well culturing for 24 h, and was infected with EV71 (MOI = 0.1). Three hrs later, EV71-infected were transfected with IgG using Liposomal Transfection Reagent DOTAP (Roche, Basel, Switzerland) as previously described [24]. Briefly, 15 μl of DOTAP was incubated with 10 μl of purified IgG (5 μg/well) for 20 min in serum-free medium. After incubation, 500 μl DMEM medium and mixture was added. Twenty-four hrs later, virus-infected cell were taken pictures and undergone three freeze-thaw circles, and were subject to determine the viral titers.

### Plaque assay

A series of 1:10 dilutions were made by mixing 15 μl of EV71-infected cell with 135 μl of DMEM. One hundred microliters of each dilution were seeded to individual wells of 24-well plate containing confluent Vero-1008 cells (2 × 10^5^ cells/well). The plate was incubated at 37 °C with 5% CO_2_ for 1 h before the layer of 2% methyl cellulose was added. After 4 days of incubation at 37 °C with 5% CO_2_, the cells were fixed in 3.7% formaldehyde and then stained with 1% crystal violet. Plaque numbers were recorded after washing the plates with tap water [25].

### RdRp-mediated RNA elongation assay in vitro

In 10 μl reaction system containing 50 mM HEPES, 75 mM KCl, 5 mM MgCl_2_, 4 mM TCEP, 300 μM NTP, and 4 μM RNA Complex, the 5 μg functional 3D^pol^ was added, and the system maintained at 22.5 °C for 30 min, finally the reaction was terminated with RAB. RNA species were resolved by 15% polyacrylamide/7 M Urea gel electrophoresis and visualized by Stains-All staining [26]. RNA elongation activity of 3D^pol^ could be assessed based on the appearance of elongated RNA band.

### In vivo evaluation of antiviral efficacy of mAbs

The protective efficacy of the mAbs was evaluated by an in vivo assay as previously described [21]. Pregnant ICR mice maintain under SFP condition. Five groups of neonatal ICR mice at 1 day of age were inoculated i.p. with 50 μg of mAb/mouse (50 μl) respectively, followed by i.p. inoculation with 3.55 × 10^6^ TCID_50_ of EV71/MAV-VR (50 μl) at 24 h later. The challenged mice were monitored daily for survival and clinical score for 16 days. Clinical scores were graded as follows: 0, healthy; 1, reduced mobility; 2, limb weakness; 3, paralysis; and 4, death. Mice that lost more than 35% of the control mice (PBS treated group) body weight were euthanized and counted as dead. All the mice were observed for 16 days and euthanized by injection with ketamine mixture. Mice were euthanized by injection (i.m.) with 15 μg/50 μl of ketamine mixture (79.3% Ketaject, 17.5% Atropine, and 3.2% Acepromazine) per gram mice body weight. The EV71/MAV-VR challenge study was approved by the ethics committee of life science and research in Wuhan Institute of Virology (WIV), Chinese Academy of Sciences (CAS) (No. WIVA09201504).

### Statistical analyses

Kaplan-Meier survival curves were used to display mortality data, and log rank analyses were performed to determine statistical significance between different groups. * indicates value of *p* < 0.05, ** indicates value of *p* < 0.01.

## Additional files

Additional file 1: **Figure S1.** Ratio of mAb heavy chain(HC)/light chain(LC). **a** Optical densitometry of antibodies were calculated with IMAGE J tool. The optical densitometry of 3A12 heavy chain was defined as 100. Each densitometry of antibody HC/LC was evaluated based on the 3A12 heavy chain. **b** Ratio of each mAb heavy chain(HC)/light chain(LC) was close to 2. (JPG 35 kb)

Additional file 2: **Figure S2.** Mean fluorescence intensity (MFI) of virus-infected cells. Vero-1008 was infected with EV71 at MOI = 0.01. At 48 h of infection, cells were harvested, followed by fixation and permeation. Virus-infected cells were incubated with mAbs, subsequently subject to FACS analysis. MFI of each mAb-treated cell was ~ 5000. MAb 5G10 (against bacterial flagellin) served as an irrelative control. (JPG 21 kb)

Additional file 3: **Figure S3.** EV71 3D^pol^-specific mAbs were unable to extracellularly neutralize virus. One hundred PFU of EV71 incubated with 5 μg of mAb, at 1 h incubation mixture was added to Vero-1008 in 24-well plate. At another 2 h incubation, culture was removed and then cells were cultured by incomplete DMEM containing 1% CMC, followed by additional culture for 3 d. Finally, cell plaques were visualized and counted after crystal violet staining. EV71 3D^pol^-specific mAbs were unable to extracellularly neutralize virus as the 5G10 irrelative control. Monoclonal antibody (mAb) D5 towards VP1 with neutralization capacity served as positive control. MAb 5G10 against flagellin acted as an irrelevant mAb control. (JPG 26 kb)

## Abbreviations

AA: Amino acid
CPE: Cytopathic effect
EV71: Enterovirus A 71
HFMD: Hand, foot and mouth disease
i.m: Intramuscular
i.p: Intraperitoneal
mAb: Monoclonal antibody
PFU: Plaque forming unit
RAB: RNA Annealing Buffer
RdRp: RNA-dependent RNA polymerase
