# Supplementary figures and images for: Monoclonal antibody against EV71 3Dpol inhibits the polymerase activity of RdRp and virus replication
Source: BMC Immunol. 2019 Jan 22;20:6. doi: 10.1186/s12865-019-0288-x (PMC6343263; doi:10.1186/s12865-019-0288-x)

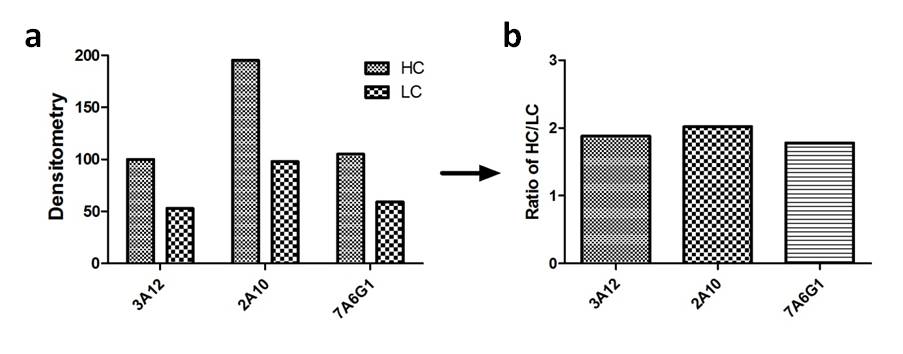

Supplement: Supplementary file 1 — Figure S1. Ratio of mAb heavy chain(HC)/light chain(LC). a Optical densitometry of antibodies were calculated with IMAGE J tool. The optical densitometry of 3A12 heavy chain was defined as 100. Each densitometry of antibody HC/LC was evaluated based on the 3A12 heavy chain. b Ratio of each mAb heavy chain(HC)/light chain(LC) was close to 2. (JPG 35 kb) [file 12865_2019_288_MOESM1_ESM.jpg]

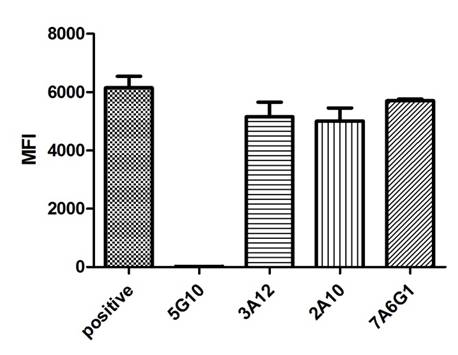

Supplement: Supplementary file 2 — Figure S2. Mean fluorescence intensity (MFI) of virus-infected cells. Vero-1008 was infected with EV71 at MOI = 0.01. At 48 h of infection, cells were harvested, followed by fixation and permeation. Virus-infected cells were incubated with mAbs, subsequently subject to FACS analysis. MFI of each mAb-treated cell was ~ 5000. MAb 5G10 (against bacterial flagellin) served as an irrelative control. (JPG 21 kb) [file 12865_2019_288_MOESM2_ESM.jpg]

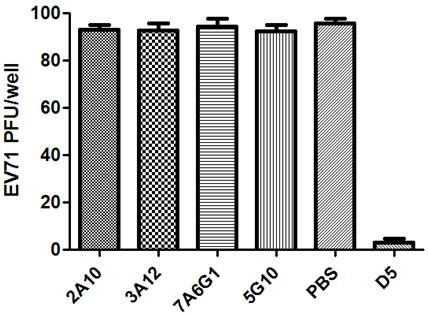

Supplement: Supplementary file 3 — Figure S3. EV71 3Dpol-specific mAbs were unable to extracellularly neutralize virus. One hundred PFU of EV71 incubated with 5 μg of mAb, at 1 h incubation mixture was added to Vero-1008 in 24-well plate. At another 2 h incubation, culture was removed and then cells were cultured by incomplete DMEM containing 1% CMC, followed by additional culture for 3 d. Finally, cell plaques were visualized and counted after crystal violet staining. EV71 3Dpol-specific mAbs were unable to extracellularly neutralize virus as the 5G10 irrelative control. Monoclonal antibody (mAb) D5 towards VP1 with neutralization capacity served as positive control. MAb 5G10 against flagellin acted as an irrelevant mAb control. (JPG 26 kb) [file 12865_2019_288_MOESM3_ESM.jpg]
